# Acknowledgement to Reviewers of *Brain Sciences* in 2014

**DOI:** 10.3390/brainsci5010001

**Published:** 2015-01-08

**Authors:** Brain Sciences Editorial Office

**Affiliations:** MDPI AG, Klybeckstrasse 64, CH-4057 Basel, Switzerland

The editors of *Brain Sciences* would like to express their sincere gratitude to the following reviewers for assessing manuscripts in 2014:

Albouy, Philippe
Aton, Sara
Augustenborg, Claudia
Bai, Xiaowen
Barutchu, Ayla
Bear, Mark F.
Bendixen, Alexandra
Bimonte-Nelson, Heather
Bjørklund, Geir
Brady, Anne Marie
Burton, Harold
Buschkuehl, Martin
Cassia, Viola Macchi
Ceroni, Fabiola
Colloc’h, Nathalie
Delogu, Franco
Dettmers, Christian
Ehrich, Marion
Eitam, Baruch
Ellerby, Lisa
Fachner, Jörg
Faraguna, Ugo
Fernández, Eduardo
Fernández-Ruiz, Javier
Futschik, Matthias E.
Garcia-Sierra, Adrian
Glaser, Bronwyn
Gold, Christian
Guo, Yanhui
Harteneck, Christian
Herholz, Sibylle C.
Hertz, U.
Ikonomidoua, Chrysanthy
Ing, Caleb
Jevtovic-Todorovic, Vesna
Jordan, Kerry E.
Kamiyama, Keiko
Key, Alexandra P. F.
Kinsley, Craig
Klein, Jochen
Kovelman, Ioulia
Kreutz, Gunter
Kuo, Chung-Hsien
Kwon, Yong Hyun
Lange, Joachim
Leeming, R. J.
Lesiuk, Teresa
Lonstein, Joseph
Looney, David
Lunardi, N.
Maher, Pamela
Martens, Marilee A.
Maxwell, Judith
Meyer, Martin
Miano, Silvia
Morone, Giovanni
Moser, Jason
Neumann, Inga D.
Oechslin, Mathias S.
Orser, Beverley A.
Ouyang, Lijing
Penhune, Virginia
Raizen, David
Renaud, Sylvie
Ron, Ricardo
Rossignol, Dan
Sagredo, Onintza
Sammler, Daniela
Sandmann, Pascale
Särkämö, Teppo
Schellenberg, E. Glenn
Schiffmann, Susanne
Schmidt, Louis A.
Scott, Olson
Shaw, Paul
Simons, Sinno HP
Simpson, Elizabeth
Singh, Leher
Slevc, L. Robert
Soriano, Sulpicio G.
Strelnikov, Kuzma
Terrando, Niccolò
Tillmann, Barbara
Vergani, Laura
Wang, Yijun
Wei, Huafeng
Wlotko, Edward
Wong, Jen D.
Xie, Z. C.
Yazdani, Ashkan
Zanto, Theodore P.
Zhou, Helen Juan

